# Sarcopenia and frailty in Parkinson’s disease: A cross-sectional analytical study

**DOI:** 10.12669/pjms.42.9.12273

**Published:** 2026-09

**Authors:** Tuba Tulay Koca, Cemile Buket Yildiz, Elif Ozel

**Affiliations:** 1 Tuba Tulay Koca Faculty of Medicine, Department of Physical Medicine and Rehabilitation, Sutcu Imam University, Faculty of Medicine, Kahramanmaraş, Turkey; 2 Cemile Buket YildizFaculty of Medicine, Department of Neurology, Sutcu Imam University, Faculty of Medicine, Kahramanmaraş, Turkey; 3 Elif OzelDepartment of Physical Medicine and Rehabilitation, Sutcu Imam University, Faculty of Medicine, Kahramanmaraş, Turkey

**Keywords:** Frailty, Geriatric syndromes, Parkinson’s disease, Sarcopenia

## Abstract

**Objective:**

Sarcopenia and frailty are common geriatric syndromes characterized by high morbidity and low physical function. Here, we aimed to analyze risk of sarcopenia and frailty in patients with Parkinson’s disease (PD).

**Methodology:**

The study was planned as cross-sectional and analytical at Physical Medicine and Rehabilitation Clinic between April and December in 2023. Sixty-two male/female patients diagnosed with PD were included in the study. SARC-F (Strength, Assistance, Rising, Climbing and Falls) (cut off ≥ 4) was used for sarcopenia risk. Muscle strength was evaluated with manual muscle strength and handgrip strength (HG). Clinical frailty scale (CFS) (<5 nonfrail) was used for frailty. Physical performance was evaluated with chair stand test and timed up and go test (TUG).

**Results:**

Total 23 female and 39 male patients with PD, with an average age of 66.2±11.4 years, were included in the study. Sarcopenia was detected in 35 (56.5%) of the participants, frailty was detected in 26 (41.9%) and malnutrition was detected in 12 (19.4%). Regardless of age (p=0.704), disease duration (p=0.157) and BMI (p=0.806); In the high sarcopenia risk group (n=35), the proportion of females sex (p=0.034) was high, while right handgrip strength (p=0.019) and left handgrip strength (p=0.003) were lower. In addition, Hoehn & Yahr staging was advanced (p=0.012), and Parkinson Disease Questionnaire (PDQ)-39 scores were higher (p<0.001) in the high sarcopenia risk group.

**Conclusion:**

Sarcopenia and frailty are commonly observed in PD and are associated with female sex, advanced disease stage and high severity, low physical performance, high kinesiophobia, higher prevalence of malnutrition, independent of age, disease duration and BMI.

## INTRODUCTION

The progressive loss of muscle strength in the elderly population is called sarcopenia. Its pathogenesis is multifactorial; In addition to age-related changes, it includes changes in muscle protein turnover, hormone levels, hormone sensitivity, pro-inflammatory status, oxidative stress and behavioral conditions (nutrition and physical activity).^1^-^4^

Parkinson’s Disease (PD) is among the common neurodegenerative diseases and its incidence doubles over the age of 30.^5^ The association of sarcopenia with age-related neurological diseases is common but has not been adequately investigated. The effects of sarcopenia and frailty on neurological diseases such as stroke, dementia and PD are not well known.

Sarcopenia and PD both involve loss of muscle mass and function. Besides different etiological and pathogenic factors, they share common associations which influence the functional status of the patient. Overlapping mechanisms include chronic inflammation, mitochondrial dysfunction, impaired muscle function, cellular and neurohormonal changes due to aging. Chronic low-grade inflammation, mitochondrial dysfunction, oxidative stress, apoptosis leading to muscle fiber loss have a role both in PD and sarcopenia. The loss of dopamin producing neurons in PD may exacerbate muscle dysfunction (control and coordination) by developing disruptions in central nervous system.^6^

In co-occurrence, they both negatively affect physical function and morbidity. Motor dysfunction in PD including bradykinesia, rigidity and postural instability, may lead to physical inactivity and a sedentary lifestyle that contribute to the development of sarcopenia. Muscle weakness and dopaminergic dysfunction, which cause motor deficits in PD, are important risk factors for sarcopenia. Both diseases are associated with impaired protein metabolism and elevated inflammatory markers, which cause muscle weakness. Elevated inflammatory cytokines, neuroinflammation (PD) and systemic inflammation (sarcopenia) may increase the loss of muscle mass and function in individuals.^7^,^8^

In the geriatric age group, there are multiple factors that affect the patient’s muscle strength and functional capacity. If a person has a chronic disease, it may be difficult to distinguish etiologies. The presence of sarcopenia is associated with negative parameters such as low functional level, falls, fractures, disease severity, frailty and mortality.^9^

Primary sarcopenia is the loss of muscle strength related to aging. Disease-related sarcopenia focuses on muscle mass rather than muscle function. The pathogenesis of sarcopenia depends on the balance of positive and negative regulators effecting muscle growth. In secondary sarcopenia, treatment of the underlying disease is required. Age-related and disease-related sarcopenia and risk factors for sarcopenia, especially risk factors such as low physical activity and sedentary life, should be described by health care professionals.^10^-^13^

In the study, we examined sarcopenia risk, frailty and related clinical parameters such as disease severity, physical performance, muscle strength and nutrition in patients with PD. The study has several strengths. We evaluated both sarcopenia risk and fraility simultaneously in patients with PD. Secondly multiple clinical relevant factors such as disease severity, stage, physical performance, nutrition, kinesiophobia, providing a multidimensional perspective. The study findings support early identification and targeted interventions in clinical practice.

## METHODOLOGY

The study was planned as a cross-sectional and analytical at Physical Medicine and Rehabilitation Clinic between April and December in 2023. Sixty men and women diagnosed with PD were included in the study with consecutive nonprobability sampling. This approach was selected because of feasibility and study criteria. Sociodemographic data of the patients, including age, sex, body mass index (BMI) and the drugs they used were recorded. SARC-F (Strength, Assistance, Rising, Climbing and Falls) screening tool was used for sarcopenia risk. Patients with Parkinson’s disease were assessed and enrolled by an experienced neurologist specializing in movement disorders according to predefined inclusion and exclusion criteria. The diagnosis of Parkinson’s disease was established using UK Parkinson’s Disease society Brain Bank diagnostic criteria. The modified Hoehn & Yahr scale and Parkinson’s Disease Questionnaire (PDQ-39)^14^ were used for disease staging and severity. Patients who had chronic other neurological disease, degenerative joint disease, cardiovascular disease, chronic gastrointestinal system problem, rheumatic disease, psychiatric disease, orthopedic trauma or surgery, which may affect nutrition, muscle strength and performance, were excluded from the study. Vascular parkinsonism, Huntington’s disease, drug-induced parkinsonism, frontotemporal dementia, multiple system atrophy and essential tremor, which are included in the differential diagnosis of PD, were excluded by detailed clinical examination, anamnesis and neuroimaging studies.

Plasma albumin and lymphocyte values were recorded from the patients’ last three month archive records. Tampa scale for kinesiophobia (TSK) was used for assessment of kinesiophobia (the fear of movement or reinjury).^15^

### Evaluation of sarcopenia:

According to the definition of European Sarcopenia Working Group (2018) criteria both low muscle mass and muscle function are necessary in sarcopenia.^16^ SARC-F scale (cut off for sarcopenia ≥ 4) was used for sarcopenia risk. Muscle quantity and quality were not measured by dual energy absorptiometry (DXA), magnetic resonance imaging (MRI), ultrasonography (US) and computed tomography (CT). We did not use the EWGSOP-2018 diagnostic criteria for sarcopenia because the purpose of the study was to assess sarcopenia risk rather than to establish a definitive diagnosis of sarcopenia. Therefore, sarcopenia risk was evaluated using the SARC-F scale. It is a validated screening tool for identifying individuals at risk of sarcopenia.

### Evaluation of muscle strength:

Muscle strength is evaluated by the Medial Research Council with manual muscle strength and hand grip strength. Measurement of handgrip strength is a simple and inexpensive method for muscle strength assessment. Handgrip strength of the hand was assessed using a handgrip dynamometer (TKK 5401 Grip strength dynamometer, Takei Corporation, Niigata, Japan) with an accuracy of ± 2%. Clinical frailty scale (CFS<5, nonfrail) is used for frailty.^17^Measurements were performed with the patient in a seated position, with the shoulder in adduction and neutral rotation, the elbow flexed at 90°, and the forearm and wrist in a neutral position. Participants were asked to squeeze the dynamometer with maximum effort for a few seconds; the test was repeated three times for each hand with intervals, and the average value was recorded.

### Physical performance tests:

The chair stand test is used to measure quadriceps muscle group strength and endurance. Timed-Up and Go test (TUG) was used for physical performance (>12 s fall risk; 20 s cut-off value).^18^

### Status of nutrition:

Prognostic nutritional index (PNI) was calculated by recording albumin and lymphocyte values from archive records (last three months). The formula PNI= serum albumin (g/dL) + [5× lymphocyte count (/mm3)] (cut off<50 malnutrition) was used.^19^

### Statistical analysis:

Statistical analyzes were performed using the Statistical Package for the Social Sciences (SPSS) 20.0 (SPSS Inc., Chicago, IL, USA) program. A power analysis was done for sampling size. Moderate effect size, a significance level of p=0.05, a power of 80%, the minimum sample size was calculated as 62 (with considering 10% loss, minimum sample size was calculated as 69). Normality distributions were made by Kolmogorov Smirnov test and histogram analysis (skewness, kurtosis). The mean, standard deviation, median, minimum, maximum, number and percentages of parameters were calculated according to their distribution. The Chi-square and Fisher’s exact test were used to assess non-numeric parameters; Independent Samples Student t test and Mann Whitney U tests were used for numeric data according to their distribution. A multiple linear regression model was used to identify potential predictor variables associated with frailty. Spearman correlation test was used for correlation analysis (N>30). A P value of < 0.05 was used for statistically significance.

### Ethical approval:

The study complied with the principles of the International Declaration of Helsinki and ethical approval was obtained from Kahramanmaraş Sutcu Imam University Clinical Research Ethics Committee (date: 06/12/2022; protocol no: 338). An informed consent form was taken from the participants.

## RESULTS

### Demographic and Clinical Results:

Total 23 female and 39 male patients with PD, with an average age of 66.2±11.4 years, were included in the study. The mean BMI was 27.9±3.4 kg/m^2^. The median duration of the disease was 30 months (minimum one month - maximum 180 months). Almost half (41.9%) of the participants were Stage-I according to the modified Hoehn &Yahr staging. High sarcopenia risk was detected in 35 (56.5%) of the participants, frailty was detected in 26 (41.9%) and malnutrition was detected in 12 (19.4%) (Table-I). Low muscle strength was detected in 17 (43.6%) of male and 3 (13%) of female patients. According to the presence of sarcopenia; independent of age (p=0.704), disease duration (p=0.157) and BMI (p=0.806); In the high sarcopenia risk group (n=35), the proportion of females sex (p=0.034) was high, while right handgrip strength (p=0.019) and left handgrip strength (p=0.003) were lower. In addition, Hoehn & Yahr staging was advanced (p=0.012), and Parkinson Disease Questionnaire (PDQ)-39 scores were higher (p<0.001). Other parameters were found similar in both groups. The drugs they used were as listed: Levodopa (n=11), pramipexole (n=17), rasagiline (n=4), levodopa and benserazide (n=8), rivastigmine (n=1).

**Table-I:** Sociodemographic and disease related data of the overall group.

	N=/%, mean±standard deviation/median (minimum-maximum)
Age (year)	66.2±11.4
Sex (female)	23 (37.1%)
BMI (kg/m^2^)	27.9±3.4
***Modified Hoehn&Yahr scale***	
1	26 (41.9%)
1.5	9 (14.5%)
2	0
2.5	14 (22.6%)
3	8 (12.9%)
4	4 (6.5%)
5	1 (1.6%)
Disease duration (months)	30 (1-180)
Parkinson Disease Questionnaire (PDQ-39)	49.1±28.8
Timed up and go test (≥20 sn)	18.2±11.9
Chair stand test (number of sit and stand up in 15 sn, cut off<5)	3.4±2
PNI (albumin g/dL/5xlymphocyte mm^3^)	52.5±5.6
Malnutrion presence (PNI<50)	12 (19.4%)
SARC-F scale (cut of ≥ 4)	3.7±2.5
High sarcopenia risk	35 (56.5%)
Clinic Frailty score (CFS) <5, nonfrail.	1.9±1.3
***Frailty presence***	
Normal	15 (24.2%)
Prefrail	19 (30.6%)
Frail	26 (41.9%)
Hand grip strength, kg In both sexes	31.3±18.7 (right), 29.1±16.6 (left)
TSK (17-68)	41.2±11.9

***Abbreviations:*** BMI: Body mass index, PNI: prognostic nutritional index, SARC-F: sarcopenia scale, CFS: Clinic frailty score, TSK: Tampa scale for kinesiophobia.

### Correlation tests results:

In Spearman correlation analysis; SARC-F value was negatively correlated with hand strength right (rho: -0.295; p=0.02), hand strength left (rho=-0.324; p=0.01), PNI (rho=-0.471, p=0.007); positively correlated with PDQ-39 (rho:0.597, p<0.001) and Frailty score (rho=0.377, p=0.003). Frailty score was negatively correlated with chair stand test (rho=-0.431, p=0.001), lymphocyte count (rho=-0.460, p=0.001) and PNI (rho: -0.482, p=0.008); positively correlated with PDQ-39 (rho=0.388, p=0.002) and TSK (rho=0.393, p=0.003).

### Regression analysis:

In multiple linear regression analysis, longer disease duration (p<0.001) and higher TSK scores (p<0.001) were found to be positive predictors of frailty, whereas BMI was negatively associated with frailty (p=0.013) (Table-II).

**Table-II:** Multiple linear regression analysis for frailty in overall group.

	B (95% Confidence Interval for B)	P
Age	0.18 (-0.17 to 0.53)	0.28
Disease duration	0.065 (0.033 to 0.075)	<0.001*
BMI	-0.137 (-0.240 to -0.035)	0.013*
Albumin	0.053 (-0.108 to 0.215)	0.48
PDQ-39	0.008 (-0.011 to 0.027)	0.38
TSK	0.116 (0.077 to 0.155)	<0.001*

***Abbreviations:*** BMI: body mass index, TSK: tampa scale for kinesiophobia, PDQ-39: Parkinson disease questionnaire-39.

* p<0.05, statistically significance.

## DISCUSSION

The exact prevalence of sarcopenia in chronic neurological diseases is unknown. In our study, we found high sarcopenia risk in female sex. We found that the prevalence of sarcopenia risk increases with disease severity and stage in patients with PD, independent of age, disease duration, and BMI. Based on this, we can say that common factors other than advanced age and BMI play a role in the pathogenesis of PD and sarcopenia. In our study, the presence of frailty was associated with low scores in physical performance tests, kinesiophobia and low lymphocyte count. The presence of kinesiophobia may be associated with frailty through the individual’s low physical activity. Both parameters sarcopenia and frailty were closely related to each other. Poor muscle strength and physical performance, associated with handgrip strength and low walking speed, cause high morbidity and mortality.

Sarcopenia, which develops secondary to neurological diseases, progresses in parallel with the clinical progression of the disease.^20^,^21^ For those with chronic neurological disease, inadequate nutritional support, neurological factors, endocrine changes and physical inactivity may cause the development of sarcopenia.^22^,^23^ In our study, malnutrition was found to be significantly associated with sarcopenia risk and frailty. Optimizing nutritional status for patients is important to prevent the development of sarcopenia. Frailty is associated with low physical performance scores.^24^,^25^ Current scientific research focuses on identifying biomarkers in the diagnosis of PD and finding effective treatments.^26^,^27^

In our study, nearly half of the patients were in the early stage. Physical performance tests in the high sarcopenia risk group were similar to the other group. Based on this, we can say that there are other factors other than sarcopenia that affect the physical function of the individual. In the meta-analysis conducted by Cai Y et al.^28^, the prevalence of sarcopenia in PD varies between 6% and 55%. In our study, the prevalence of high sarcopenia risk was 56.5%; the prevalence of frailty was 41.9%. According to the study by Yuan S et al.^29^ sarcopenia affects 10% to 16% of the elderly population worldwide. The higher risk of sarcopenia and the presence of frailty in patients with PD compared to the general population suggest an association between the two conditions. In systematic metanalysis by To TL et al.^30^ frailty prevalence is 46.3% by the Clinical Frailty Scale. In the metanalysis by Hart A et al.^31^, sarcopenia increases in both PD and Parkinson-related diseases and is related to disease severity. This association emphasizes the importance of sarcopenia screening in parkinsonian groups. In a similar study conducted by Da Luz MCL et al.^32^ the prevalence was 19.5% and was found to be associated with age, poor performance and nutritional status. SARC-F scale was also found to be associated with Parkinson’s severity.^33^ In our study, SARC-F was positively correlated with disease stage and severity. As disease severity and stage increase, the risk of sarcopenia increases accordingly.

As symptoms progress in PD, there is difficulty in hand functions and walking and a tendency to fall. Baseline motor and cognitive impairment are predictive of more rapid motor retardation and disability. Both sarcopenia and PD are related to an increased risk of falling in old age which is associated with functional disability, frailty and mortality. An additional difficulty is the side effects associated with levodopa. In these patients, balance, posture, hand and arm functions and gait should be evaluated.^34^ Falls in the older age group causes kinesiophobia, serious injuries, fractures, inadequacy in daily living activities, less confidence and a less active lifestyle in older adults.^34^ In our study, kinesiophobia levels were similar between participants with and without a high risk of sarcopenia; however, kinesiophobia was higher in the frail group.

In PD, there is a fear of falling due to body flexion posture and trunk instability, which may cause kinesiophobia in patients. In our study, we found a higher rate of low muscle weakness in male patients. This situation can be explained by the decrease in testosterone hormone and has a negative impact on the risk of falling and loss of physical function in patients with PD.^35^

Due to aging, we see cumulative erosion in many systems, especially the immune system.^36^ Our study supports that high frailty and low lymphocyte count, regardless of age, may also play a role in other mechanisms. In the study conducted by Kaya T et al., it was observed that the neutrophil-lymphocyte ratio was significantly related to nutrition. Nutrition may play a role in the association of the immune system and frailty.^37^

The single-center design, sample imbalance of sexes in the groups and the lack of evaluation of quality of life and balance are the weaknesses of the study. Almost half (41.9%) of the participants were stage-I according to the modified Hoehn & Yahr staging. The study group consisted mostly of early stage. The cross-sectional analytical design of the study limits the ability to establish causal relationships.

## CONCLUSION

High sarcopenia risk and frailty are common in patients with PD and are independently associated with female sex, advanced disease stage and severity, lower physical performance, higher levels of kinesiophobia, and malnutrition, independent of age, disease duration, and BMI. The coexistence of these conditions may be associated clinically, and linked to worse physical performance and functional status in patients with PD. Early evaluation of sarcopenia, frailty and related factors (disease severity, nutrition, BMI, immune system, muscle strength) are may be important for morbidity, physical inactivity, falls and disability in patients with PD.

### Disclosure:

The AI was not use to generate, analyze or interpret primary research data.

### Authors’ contribution:

**TTK:** Conception, design, acquisition, analysis, interpretation of data, final approval.

**CBY:** Hypothesis, critical review of the paper.

**EÖ:** Data collection.

All authors have read and approved the final version. They are also responsible for the integrity and accuracy of the study.
